# Candidate proteomic biomarkers for three genogroups of the swine pathogen *Streptococcus suis* serotype 2

**DOI:** 10.1186/s12866-015-0401-0

**Published:** 2015-04-04

**Authors:** Christo Atanassov, Laetitia Bonifait, Marylise Perivier, Marcelo Gottschalk, Daniel Grenier

**Affiliations:** Laboratoire de Bactériologie-Hygiène, Centre Hospitalier Universitaire de Poitiers, Poitiers, France; EA 4331 LITEC, Pôle Biologie-Santé, Université de Poitiers, Poitiers, France; Groupe de Recherche en Écologie Buccale, Faculté de Médecine Dentaire, Université Laval, 2420 de la Terrasse, Quebec City, QC G1V 0A6 Canada; Groupe de Recherche sur les Maladies Infectieuses du Porc, Faculté de Médecine Vétérinaire, Université de Montréal, Saint-Hyacinthe, QC Canada; Centre de Recherche en Infectiologie Porcine et Avicole (CRIPA), Fonds de Recherche du Québec - Nature et Technologies (FRQNT), Saint-Hyacinthe, QC Canada

**Keywords:** Proteomic, SELDI, *Streptococcus suis*, Swine infection

## Abstract

**Background:**

*Streptococcus suis*, more specifically serotype 2, is a major swine pathogen and an emerging zoonotic agent that causes severe infections such as meningitis, endocarditis, and septicemia. In this study, surface-enhanced laser desorption/ionization time-of-flight mass spectrometry (SELDI) was used to investigate the protein expression profiles of 45 strains of *S. suis* serotype 2 that had previously been clustered by multilocus sequence typing (MLST) into three sequence types (ST1, ST25, and ST28) (n = 15 for each ST).

**Results:**

The SELDI data were analyzed using the univariate Mann–Whitney and Kruskal-Wallis tests and multivariate statistical methods (heatmap/hierarchical clustering). The heatmap identified 136 cell proteins, and hierarchical clustering provided a 100% correct classification of all fifteen ST1 and ST25 strains and thirteen of the fifteen ST28 strains (87% correct). The univariate statistical analyses of the SELDI protein expression profiles identified nine significant proteins that discriminated the strains of the three STs of *S. suis.* Of these proteins, two were overexpressed in ST1 (5958 Da and 10249 Da), four in ST25 (5989 Da, 6646 Da, 7421 Da, and 9825 Da), and three in ST28 (4516 Da, 7833 Da, and 9342 Da). Two of the proteins associated with the ST28 strains (p4516 and p9342) were purified and were identified as a putative ABC transporter and a nucleoid-DNA-binding protein, respectively.

**Conclusions:**

SELDI analysis of 45 strains of *S. suis* allowed to identify nine statistically significant proteins that can be specifically correlated with either ST1, ST25 or ST28. The possible involvement of the overexpressed proteins in the pathology of *S. suis* infections will require further investigation.

**Electronic supplementary material:**

The online version of this article (doi:10.1186/s12866-015-0401-0) contains supplementary material, which is available to authorized users.

## Background

*Streptococcus suis*, more particularly serotype 2, is a major swine pathogen and an emerging zoonotic agent. In Western countries it mainly affects individuals with occupational exposure to pigs who are in direct contact with farm animals or who handle pork meat and carcasses [1,2]. Zoonotic infections caused by *S. suis* are encountered more frequently in East Asia (China, Vietnam, Cambodia, Thailand, Hong Kong) where the general population is also at risk due to backyard food production systems, wet markets, and/or consumption of raw pork meat/blood [1-3]. Porcine and human infections can be severe, with meningitis, endocarditis, and septicemia as possible clinical outcomes [3,4]. In humans, mortalities ranging from 5% to 20% have been reported [1,5].

*S. suis* is a Gram-positive bacterium with cell wall antigenic determinants somehow related to Lancefield group D streptococci [4]. Currently, 33 serotypes have been described based on the composition of their capsular polysaccharides [2]. While the serotype distribution varies depending on the geographical origin of the strains, *S. suis* serotype 2 is considered the most pathogenic and the most prevalent capsular type among diseased pigs and humans [2,6,7]. Multilocus sequence typing (MLST) has shown that *S. suis* serotype 2 strains can be divided into different sequence types (STs) [8]. Closely related STs are grouped in ST complexes, and several dominate the *S. suis* population, including ST1, ST16, ST25, ST28, and ST147. Most invasive strains causing human outbreaks in Europe and Asia are ST1 strains [9-11] or, at least, belong to the ST1 and ST16 complexes [8,12-14]. This is in agreement with the recent study by Fittipaldi et al. [15], who showed, using a mouse infection model, that European ST1 strains are highly virulent while North American ST25 strains display moderate virulence and North American ST28 strains are weakly virulent.

The reasons for these differences in virulence are not well understood [15] while the discovery of novel candidate virulence factors that are either produced exclusively or overexpressed by ST1 strains is a major theoretical and practical issue. The search for putative *S. suis* virulence factors has been conducted using genomic [16-22] and proteomic tools [23-31]. Proteomic studies of *S. suis* are based on the initial separation of bacterial proteins by two-dimensional polyacrylamide gel electrophoresis (2D PAGE) followed by the enzymatic digestion of selected protein bands and the identification of the resulting peptides by mass spectrometry combined with bio-informatics analyses of databases of sequenced genomes from this taxon. While proteomic methods have made a major contribution, they suffer from limitations inherent to 2D electrophoresis, which often fails to detect some proteins, especially those with small molecular weights or extreme isoelectric points. These limitations can be overcome using surface-enhanced laser desorption/ionization time-of-flight mass spectrometry (SELDI-TOF-MS; SELDI ProteinChip), a medium-throughput technology that allows protein expression levels in hundreds of samples in a single experiment to be compared [32,33]. We used this approach to analyze 45 *S. suis* serotype 2 strains and found nine statistically significant proteins correlated to the three STs that had previously been identified by MLST (ST1, ST25, and ST28). Moreover, two of these biomarkers were purified and identified.

## Methods

### Bacterial strains

Forty-five *S. suis* serotype 2 strains isolated from diseased pigs or humans from different countries were included in the SELDI analysis. They were either ST1, ST25, or ST28 strains (see Table 1) based on previous MLST analyses ([15], unpublished data).

**Table 1:** **Characteristics of** ***S. suis*** **strains serotype 2 used in the SELDI analysis**

**Strains**	**ST**	**Country**	**Province/State**	**Infection**
**P1/7**	1*	Europe	NA****	Meningitis
**MNCM01**	1	Thailand	Chiang Mai	Endocarditis
**MNCM06**	1	Thailand	Chiang Mai	Meningitis
**MNCM16**	1	Thailand	Chiang Mai	Meningitis
**MGGUS2**	1	United States	Wisconsin	Meningitis
**MGGUS3**	1	United States	Iowa	Meningitis
**NIAH11433**	1	Japan	NA	Meningitis
**DAT261**	1	Japan	Gunma	Pneumonia
**DAT264**	1	Japan	Gunma	Meningitis
**DAT229**	1	Japan	Aichi	Endocarditis
**P 517/03 P4**	1	Argentina	Buenos Aires	Meningitis
**P 477/03 P1**	1	Argentina	Cordoba	Meningitis
**P 613/05 P3**	1	Argentina	Buenos Aires	Meningitis
**31533**	1	France	Brittany	Meningitis
**SS166**	1	France	Brittany	Meningitis
**1043248**	25**	Canada	Quebec	Meningitis
**1043629**	25	Canada	Quebec	Pneumonia
**1044423**	25	Canada	Ontario	NA
**1053253**	25	Canada	Manitoba	Pneumonia
**1085543**	25	Canada	Quebec	Meningitis
**MNCM04**	25	Thailand	Chiang Mai	Meningitis
**MNCM10**	25	Thailand	Chiang Mai	Septicemia
**MNCM24**	25	Thailand	Chiang Mai	Endocarditis
**MNCM26**	25	Thailand	Chiang Mai	Meningitis, endocarditis
**MNCM51**	25	Thailand	Chiang Mai	Septicemia
**MGGUS4**	25	United States	Iowa	Septicemia
**1102864**	25	Canada	Quebec	Septicemia
**1102337**	25	Canada	Quebec	Meningitis
**LPH4**	25	Thailand	Lamphun	Septicemia
**LPH12**	25	Thailand	Lamphun	Septicemia
**1054471**	28***	Canada	Manitoba	Meningitis
**1088563**	28	Canada	Quebec	Meningitis
**1097205**	28	Canada	Ontario	Meningitis
**1057906**	28	Canada	Saskatchewan	Meningitis
**MNCM43**	28	Thailand	Chiang Mai	Endocarditis
**MGGUS9**	28	United States	Oklahoma	Endocarditis
**MGGUS10**	28	United States	Illinois	Pneumonia
**MGGUS11**	28	United States	Virginia	Pneumonia
**MGGUS12**	28	United States	Iowa	Pneumonia
**DAT242**	28	Japan	Ibaraki	Meningitis
**DAT245**	28	Japan	Ibaraki	Meningitis
**DAT246**	28	Japan	Ibaraki	Septicemia
**1084708**	28	Canada	Ontario	Septicemia
**PAH-17**	28	United States	Minnesota	Meningitis
**DAT251**	28	Japan	Ishikawa	Septicemia

*, All ST1 strains are *mrp*+, *ef*+, *sly*+; **, All ST25 strains are *mrp*-, *ef*-, *sly*-; ***, All ST28 strains are *mrp*+, *ef*-, *sly*-; ****, Not available.

### Preparation of bacterial cells

All *S. suis* strains were grown in parallel at 37°C in 10 ml of Todd-Hewitt broth (BBL Microbiology Systems, Cockeysville, MD, USA) until they reached an optical density (660 nm) in the range of 0.4 to 0.5 corresponding to the mid-log phase. The bacterial cells were harvested by centrifugation at 3,000 × *g* for 10 min at 4°C. The cell pellets were washed three times in 50 mM phosphate-buffered saline (pH 7.4) (PBS) supplemented *ex tempore* with 2 mM 4-(2-aminoethyl)benzenesulfonyl fluoride hydrochloride (AEBSF; Sigma-Aldrich Canada, Oakville, ON, Canada). After centrifugation at 3,000 × *g* for 10 min at 4°C, the cell pellets were immediately stored at −80°C until used.

### Protein extraction

The frozen cell pellets were thawed and were individually suspended in 1 ml of lysis buffer (16 mM Na_2_HPO_4_, 4 mM NaH_2_PO_4_, 150 mM NaCl, 1% Triton X-100) supplemented with cOmplete protease inhibitor cocktail^®^ (Roche Diagnostics, Laval, QC, Canada). The suspensions were transferred into FastPROTEIN BLUE tubes and were homogenized using a FastPrep apparatus (MP Biomedicals, Solon, OH, USA). The suspensions were homogenized using six cycles (40 s each) at power setting 6, with a 5-min cooling period on ice between each cycle. The homogenates were then centrifuged at 15,000 × *g* for 15 min, and the supernatants were divided into aliquots and stored at −80°C until used.

### ProteinChip array processing

Two types of ProteinChip ion-exchange arrays (Q10 and CM10; Bio-Rad Laboratories, Mississauga, ON, Canada) were assembled in a 96-well bioprocessor (Bio-Rad Laboratories) and were preactivated for 30 min with their respective buffers (100 mM Tris–HCl, pH 9.0, and 100 mM sodium acetate, pH 4.0, respectively). Binding buffers (180 μl) for the respective arrays were then mixed with 20 μl of protein extract adjusted to a final protein concentration of 0.5 mg/ml, and the mixtures were incubated for 60 min. All the protein samples were tested in duplicate. After two washes with binding buffer and one quick rinse with HPLC-grade water the spots were loaded twice with 1 μl of a saturated solution of sinapinic acid dissolved in 50% acetonitrile (ACN)/0.5% trifluoroacetic acid (TFA) (v/v). All the steps were carried out at room temperature (18-20°C) using a Micromix-5 platform shaker and a Biomek 3000 robot-pipetting workstation (Beckman-Coulter France, Villepinte, France). The arrays were processed using a PCS 4000 ProteinChip Reader (Bio-Rad Laboratories) programmed in positive ion mode with an ion acceleration potential of 20 kV.

### Spectra processing and statistical analysis

Once the spectra were calibrated and normalized using the total ion current method, clusters of peaks with the same mass were defined using the following settings: S/N (first pass) ≥5, minimum peak threshold: 20%, mass error: 0.3%, S/N (second pass) ≥2. Three types of computer-generated statistics performed using ProteinChip Data Manager 3.0.7 software (Bio-Rad Laboratories) were used to analyze the data (the non-parametric Mann-Whitney U test, the Kruskal-Wallis H test, and the heatmap/hierarchical clustering method).

### Protein purification and identification

The protein extracts were dialyzed overnight at 4°C against a 1,000-fold volume of 20 mM Tris-HCl (pH 9.5). They were then fractionated by ion-exchange chromatography (IEX) using HiTrap Q HP columns (GE Healthcare Life Sciences, Velizy-Villacoublay, France) as described previously [34]. IEX fractions containing the target proteins were further separated by reversed-phase high pressure liquid chromatography (RP-HPLC; GE Healthcare Life Sciences) according to Lanotte et al. [34]. The RP-HPLC fractions were concentrated 20-fold in a vacuum centrifuge (miVac, Genevac, Ipswich, England) to a final volume of ~50 μl. Thereafter, 3 μl of the concentrate was spotted on gold arrays (Bio-Rad Laboratories) and was analyzed in the MALDI mode using a SELDI PCS 4000 apparatus and the following acquisition protocol: focus mass 10,000; laser energy 3,000; matrix attenuation 2,500; partition 1/1; 20 shots. The fraction containing the target protein was dried completely in a vacuum centrifuge and was then reconstituted in tricine sodium dodecyl sulfate (SDS) sample buffer containing NuPAGE reducing agent (Invitrogen SARL, Cergy, France). The mixture was divided into two samples that were heated at 40°C for 30 min and separated in parallel on 1D home-cast Tris-Tricine gels (18%T/6%C) at 30 V constant for 1 h followed by 60 mA constant for an additional 15 h. Three lanes (two lateral and one in the middle of the gel) were loaded with pre-stained molecular weight (MW) markers, which served to indicate the approximate position of the target proteins in the unstained gel. One-mm-thick gel slices covering two adjacent lanes containing the same protein were excised. Each gel slice was further divided into two equal parts corresponding to one separate lane. One of the slices was passively eluted to extract the target protein as described previously [35]. Once the mass of the passively eluted target protein was confirmed on the gold array, the protein in the corresponding second slice was trypsin digested and LC-MS/MS microsequenced as described previously [34].

### Database search and processing of results

Data were searched using SEQUEST through a Bioworks 3.1.1 interface (ThermoFinnigan, San Jose, CA, USA) against a subset of the NCBI non-redundant database restricted to *S. suis* entries (http://www.ncbi.nlm.nih.gov/genome/genomes/199). Peak lists were extracted using extract-msn (BioWorks 3.3.1 Thermo Scientific) using the default settings. DTA files were generated from MS/MS spectra that reached a minimal intensity (n = 100) and a sufficient number of ions (n = 5). The DTA files allowed several MS/MS spectra corresponding to the same precursor ion with a tolerance of 50 ppm to be averaged. Spectra from peptides with molecular masses between 600 Da and 4,500 Da were retained. The search parameters were as follows: the mass accuracy of the monoisotopic peptide precursor was set at 10 ppm and the mass accuracy of the peptide fragments was set at 0.5 amu. Only b-ions and y-ions were considered for the mass calculations. The oxidation of methionine (+16 Da) was considered as a variable modification. Two missed trypsin cleavages were allowed. Only peptides with Xcorr values higher than 2.0 (double charge), 2.5 (triple charge), and 3.0 (more than 3 charges) were retained. In all cases, the peptide p-value was required to be below 0.001 and the DeltaCn value to be above 0.1. All protein identifications were based on the detection of a minimum of two distinct peptides. No false positives were detected using these parameters. Shared peptides were only counted for the proteins that had the most matching peptides.

## Results

The protein expression profiles of 45 *S. suis* serotype 2 strains obtained on two types of ProteinChip arrays (CM10 and Q10) were submitted to multivariate heatmap/hierarchical clustering and univariate analyses. Hierarchical clustering was based on two main spectral features of each detected protein: a qualitative feature (m/z or mass-to-charge ratio; here z = 1, which means that m/z directly reflects the mass [expressed in Daltons]) and a semi-quantitative feature (intensity, expressed in μA per laser pulse). Hierarchical clustering of the patterns was generated with respect to the following criteria: (i) sequence type (ST1 *versus* ST25 *versus* ST28), (ii) continent of origin (Far East Asia, including Japan and Thailand *versus* North America, including Canada and USA), (iii) country of origin (Canada *versus* Japan *versus* Thailand *versus* USA), and (iv) type of infection and pathological lesion (Group A - endocarditis/pneumonia/septicemia *versus* Group B - meningitis).

**Figure 1:**
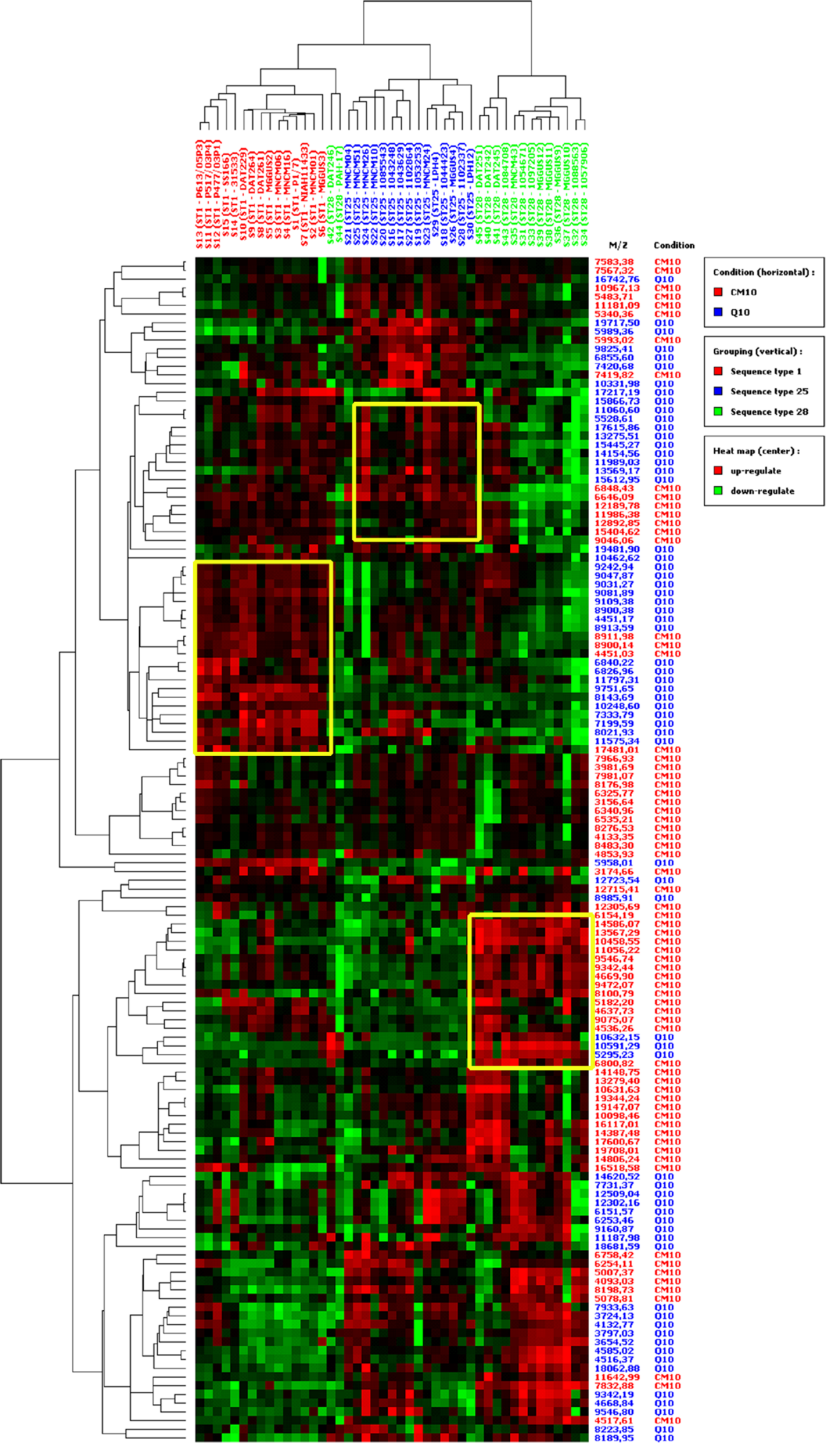
**Heatmap/hierarchical clustering of 136 proteins of 45** ***S. suis*** **strains discriminated by MLST into three sequence type (ST) groups: ST1 (n = 15), ST25 (n = 15), and ST28 (n = 15).** The clusters were obtained by combining the average intensity values of all samples tested in duplicate on CM10 and Q10 ProteinChip arrays (acquisition protocol 1). The consecutive numbers of each strain used in the SELDI expression difference mapping (EDM) are indicated above the image (S1-S15 for the ST1 group (in red); S16-S30 for the ST25 group (in blue), and S31-S45 for the ST28 group (in green) followed by the respective sequence types and original names of the *S. suis* strains (in brackets). The protein masses detected on the CM10 (red) and Q10 (blue) ProteinChip arrays are indicated on the right side of the image. Specific EDM conditions: First pass: peak S/N ≥ 5, valley depth S/N ≥ 3, minimal peak threshold – 20% for all spectra; second pass: peak S/N ≥ 2, valley depth S/N ≥ 2; third pass: adding estimated (missing) peaks to complete the clusters, clustered mass window width – 0.1%, autocentroid marks on peaks, M/Z range of analysis (z = 1): 3000–20000 Da.

While the clustering of *S. suis* protein profiles by continent, country, and pathology was unsuccessful (see Additional file 1: Figure S1, Additional file 2: Figure S2 and Additional file 3: Figure S3), the clustering of *S. suis* protein profiles by ST generated a very high level of correct classification (Figure 1). The detailed view of the heatmap on Figure 1 shows 136 proteins in the three groups of *S. suis* strains defined by ST (ST1, ST25, and ST28). Interestingly, hierarchical clustering provided a 100% correct classification of all fifteen ST1 and ST25 strains and thirteen of the fifteen ST28 strains (87% correct classification).

Some combinations of biomarkers (arbitrarily delimited by yellow- and grey-bordered rectangles on Figure 1) made it possible to delineate the groups from each other. However, none of the combinations was completely homogenous in terms of over-expression (or up-regulation) or under-expression (or down-regulation) (red and green boxes in the respective rectangles). Another practical consideration was that the number of candidate biomarkers turned out to be too large to be of use for further purification and identification in academic laboratories such as ours. In addition, while multivariate clustering has the advantage of providing a global overview of the profiles, it does not allow a precise quantification of expression. In practice, the usual colors of the rectangles on the heatmaps (red standing for over-expression or up-regulation, green for under-expression or down-regulation, and black for no difference) are nuanced as more or less dark.

**Figure 2:**
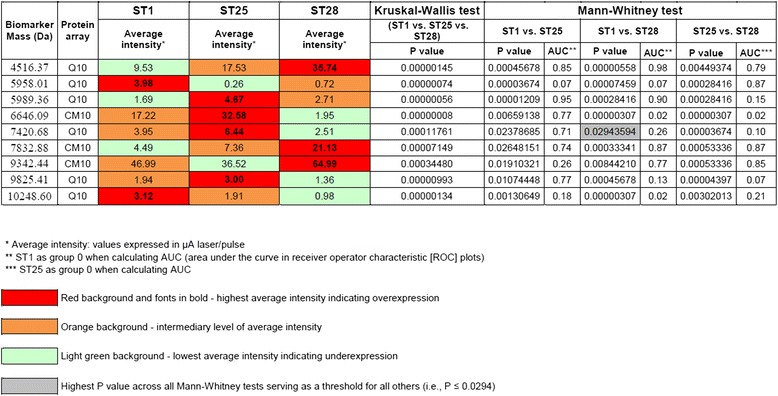
**Candidate biomarkers of** ***S. suis*** **selected by univariate analysis of two or three groups of strains divided by sequence type.**

The same spectra were analyzed by the Mann–Whitney (applicable to two groups of data sets only) and Kruskal-Wallis (applicable to more than two groups; in our study - three or four) tests to limit the number of the candidate biomarkers and to evaluate their expression levels more precisely while trying to select the most statistically discriminating ones for further purification and sequencing. The statistical comparisons were made point-by-point, that is, for candidate biomarkers with the same mass whose statistically significant variations in intensity were calculated either for *S. suis* groups defined by their ST or other criteria such as strain origin and pathology. Four selection criteria with respect to the *S. suis* strains that were discriminated by their ST (Figure 2) as well as by continent, country of origin, or pathology (Additional file 4: Table S1, Additional file 5: Table S2 and Additional file 6: Table S3) were compared using a multivariate analysis. However, unlike the multivariate analysis where discriminated protein profiles were correlated only with the ST of the *S. suis* strains, the univariate Mann–Whitney and Kruskal-Wallis analyses revealed candidate biomarkers in all cases. It should be noted that only the most statistically significant candidate biomarkers in Figure 2 and in Additional file 4: Table S1, Additional file 5: Table S2 and Additional file 6: Table S3 are presented (9 in Figure 2; 24, 30, and 12 in Additional file 4: Table S1, Additional file 5: Table S2 and Additional file 6: Table S3, respectively). Figure 2 shows the nine most discriminating proteins found in the *S. suis* strains divided by ST. Of these, two were overexpressed in ST1 (5,958 Da and 10,249 Da), four in ST25 (5,989 Da, 6,646 Da, 7,421 Da, and 9,825 Da), and three in ST28 (4,516 Da, 7,833 Da, and 9,342 Da). Interestingly, only two of the biomarkers in Figure 2 were found statistically significant when all strains were compared by combining ST and type of pathology in two groups: group A (heart/lung/sepsis) and group B (meningitis/brain). The first is p5958, which was overexpressed in the *S. suis* ST28 strains (Figure 2), as well as in strains isolated from pigs/humans with meningitis (Additional file 6: Table S3). The second is p5989, which was overexpressed in the *S. suis* ST1 strains (Figure 2) and in strains isolated from pigs/humans with cardiac and pulmonary pathologies or sepsis (Additional file 6: Table S3).

As a next step, we tried to purify and identify the most promising candidate biomarkers. They were purified using several purification steps, including IEX, RP-HPLC, and 1D SDS-PAGE. All the fractions obtained throughout the purification procedure were systematically tested for the presence of target proteins by SELDI (IEX fractions), MALDI (RP-HPLC fractions, and passive elution (1D SDS-PAGE gel slices). The purified proteins were trypsinized, identified by LC-MS/MS, and submitted to an *in silico* comparison of the masses of the peptides to the masses in the proteome database of 19 *S. suis* reference strains with completely sequenced genomes and 75 strains from the same taxon whose genomes are currently being sequenced (http://www.ncbi.nlm.nih.gov/genome/genomes/199). We eventually succeeded in identifying two of the proteins (p4516 and p9342) as a putative ATP-binding cassette (ABC) transporter and a nucleoid-DNA-binding protein, respectively.

## Discussion

Compared to studies that are usually performed on a limited number of bacterial strains, population-based studies seem to be a more appropriate approach for identifying species-specific biomarkers. Our study on a population of 45 *S. suis* serotype 2 strains is thus more representative of species whose main genotypes have already been defined by MLST. The search for candidate biomarkers has benefited from the advent of mass spectrometry, which has also proven to be a promising and reliable approach for classifying bacteria. Several techniques are currently being used, including MALDI-TOF-MS, LC-ESI-MS, and SELDI-TOF-MS [32-35]. When applied to the characterization of complex protein samples (bacterial extracts, for example), approximately 100 proteins can be detected by MALDI and SELDI while more than 500 proteins can typically be detected by LC-ESI-MS. However, LC-ESI-MS has the reputation of being labor intensive and time consuming and of requiring multiple replicates [36]. In our study, we used SELDI in which proteins and their fragments are characterized by their *m/z*, and their abundance is estimated in a semi-quantitative manner by the level of protein expression. The limitations of the present study arise from the fact that it does not take into consideration extracellular proteins as well as cell proteins that are not expressed under the bacterial growth conditions used. In addition, SELDI is more adapted for proteins with a molecular mass of less than 30 kDa.

The EDM of proteins based on their relative abundance (as deduced from measured values of μA laser/pulse) was done in extracts from bacterial cells grown in parallel under the same culturing condition, without any specific induction of protein expression. All tested 45 *S. suis* strains were divided in three groups by sequence type and the level of average protein expression was evaluated in a comparative way, within the frame of these three STs. It is noteworthy that the level of expression of a particular protein, measured in one ST group of fifteen strains, could in fact represent a normal level for this ST, but be considered as high and/or low compared to the two other ST groups.

Two peptides that were overexpressed in ST28 strains (compared to those of ST1 and ST25 groups) were purified and identified, p4516 and p9342. The first one, p4516, was a fragment of an uncharacterized protein of *S. suis, i.e.* “hypothetical protein Ssui0_06242 [*Streptococcus suis* 05HAS68]”. Its BLAST analysis revealed sequence homology (ca. 40% identity) with a family of ATP-dependent transmembrane proteins involved in the translocation of substrates across the bacterial membrane. Interestingly, except for *S. suis* 05HAS68 where this putative ABC transporter was identified, sequence homologies were all related to ABC transporters of other streptococci taxa, such as *S. agalactiae* and *S. oralis* (Additional file 7: Table S4). The second one, p9342, was a fragment of a putative nucleoid DNA-binding protein, which may play a role in genome maintenance and gene expression regulation. BLAST analysis showed that this nucleoid DNA-binding protein is very highly conserved across different *S. suis* clinical isolates (100% identity in 17 *S. suis* strains), and, to a lesser extent, in other streptococci and lactobacilli (Additional file 7: Table S4). Further studies are required to determine why these proteins are over-expressed by less virulent strains of *S. suis*. One hypothesis is that they may be the products of anti-virulence genes, which are known to interfere with bacterial virulence [37]. For instance, Zhao et al. [38] identified the ArgT protein as an anti-virulence factor since it interferes with the virulence of *Shigella flexneri* by transporting specific amino acids or by modulating the expression of the protease HtrA.

## Conclusions

In summary, SELDI analysis of 45 strains of *S. suis* allowed to identify nine statistically significant proteins that can be specifically correlated with either ST1, ST25 or ST28. The possible involvement of the overexpressed proteins in the pathology of *S. suis* infections will require further investigation.

## Additional files

Additional file 1: Figure S1. Protein profiles of *S. suis* strains divided by continent of origin: Far East (Japan and Thailand) *versus* North America (Canada and USA). Additional file 2: Figure S2. Protein profiles of *S. suis* strains divided by country of origin: Canada *versus* Japan *versus* Thailand *versus* USA. Additional file 3: Figure S3. Protein profiles of *S. suis* strains divided by type of infection/pathological lesion: group A (endocarditis/pneumonia/septicemia) *versus* group B (meningitis). Additional file 4: Table S1. Candidate biomarkers of S. suis selected by univariate analysis (Mann-Whitney test): comparison by continent of origin. Additional file 5: Table S2. Candidate biomarkers of S. suis selected by univariate analysis (Kruskal-Wallis test) of 4 groups of strains divided by country of origin. Additional file 6: Table S3. Candidate biomarkers of S. suis selected by univariate analysis (Mann-Whitney test) of two groups of strains divided by type of pathological lesion. Additional file 7: Table S4. Essential sequence and BLAST data of the two proteins identified through p4516 (putative ABC transporter) and p9342 (nucleoid-binding protein).
