# Supplementary figures and images for: Candidate proteomic biomarkers for three genogroups of the swine pathogen Streptococcus suis serotype 2
Source: BMC Microbiol. 2015 Apr 4;15:84. doi: 10.1186/s12866-015-0401-0 (PMC4450453; doi:10.1186/s12866-015-0401-0)

## Slide 1
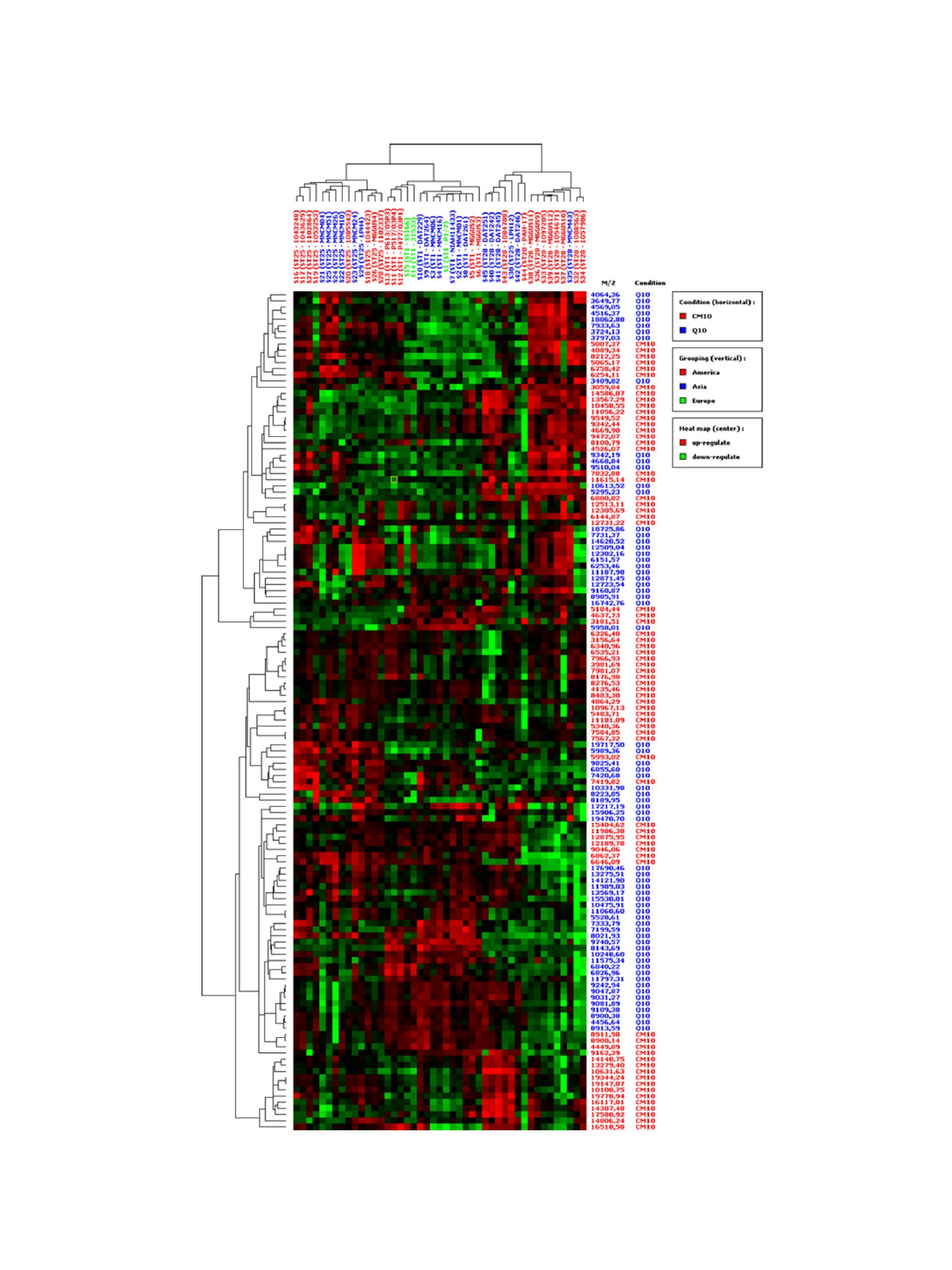

Supplement: Additional file 1: Figure S1. — Protein profiles of S. suis strains divided by continent of origin: Far East (Japan and Thailand) versus North America (Canada and USA). [file 12866_2015_401_MOESM1_ESM.pptx]

## Slide 1
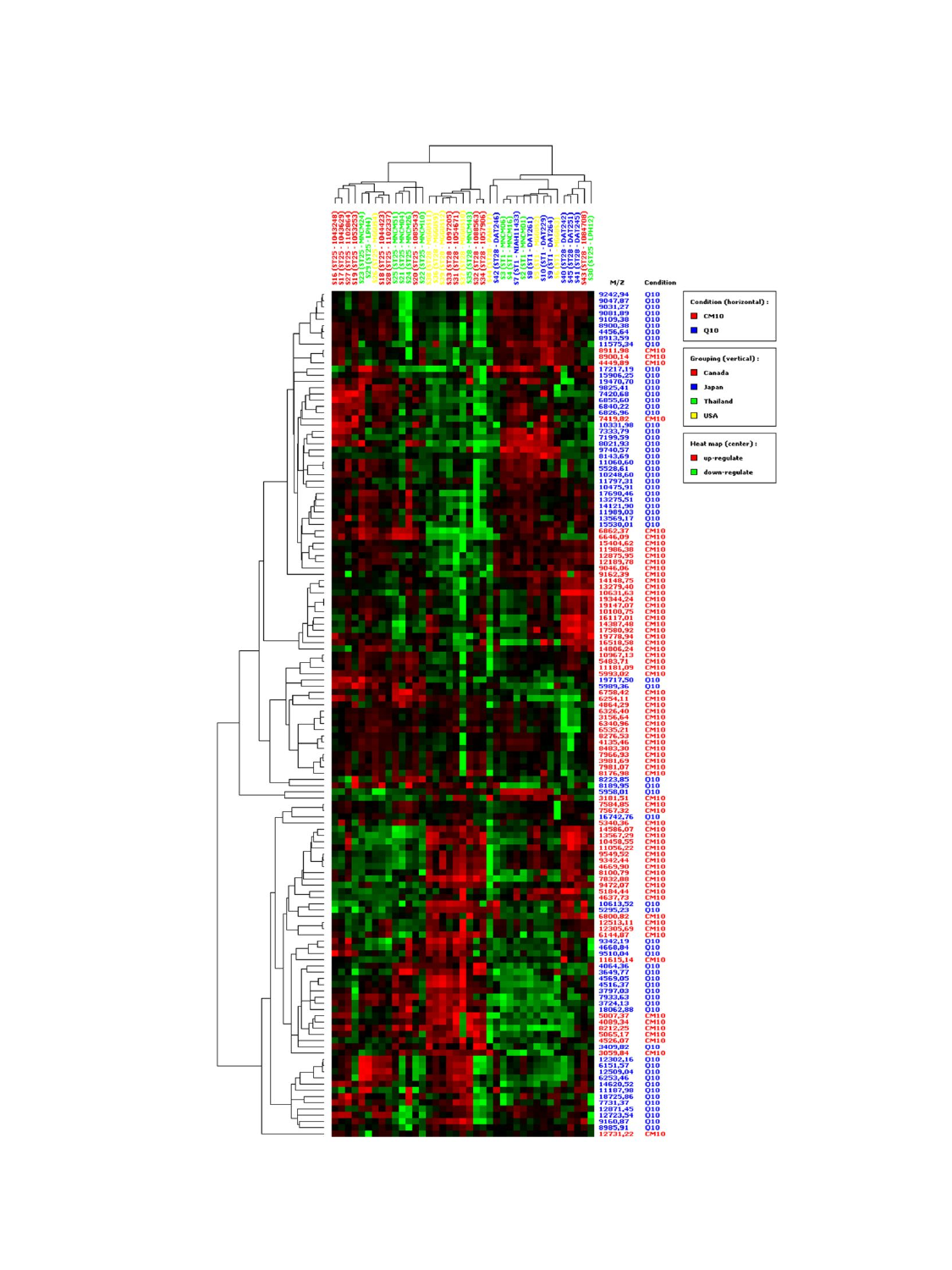

Supplement: Additional file 2: Figure S2. — Protein profiles of S. suis strains divided by country of origin: Canada versus Japan versus Thailand versus USA. [file 12866_2015_401_MOESM2_ESM.pptx]

## Slide 1
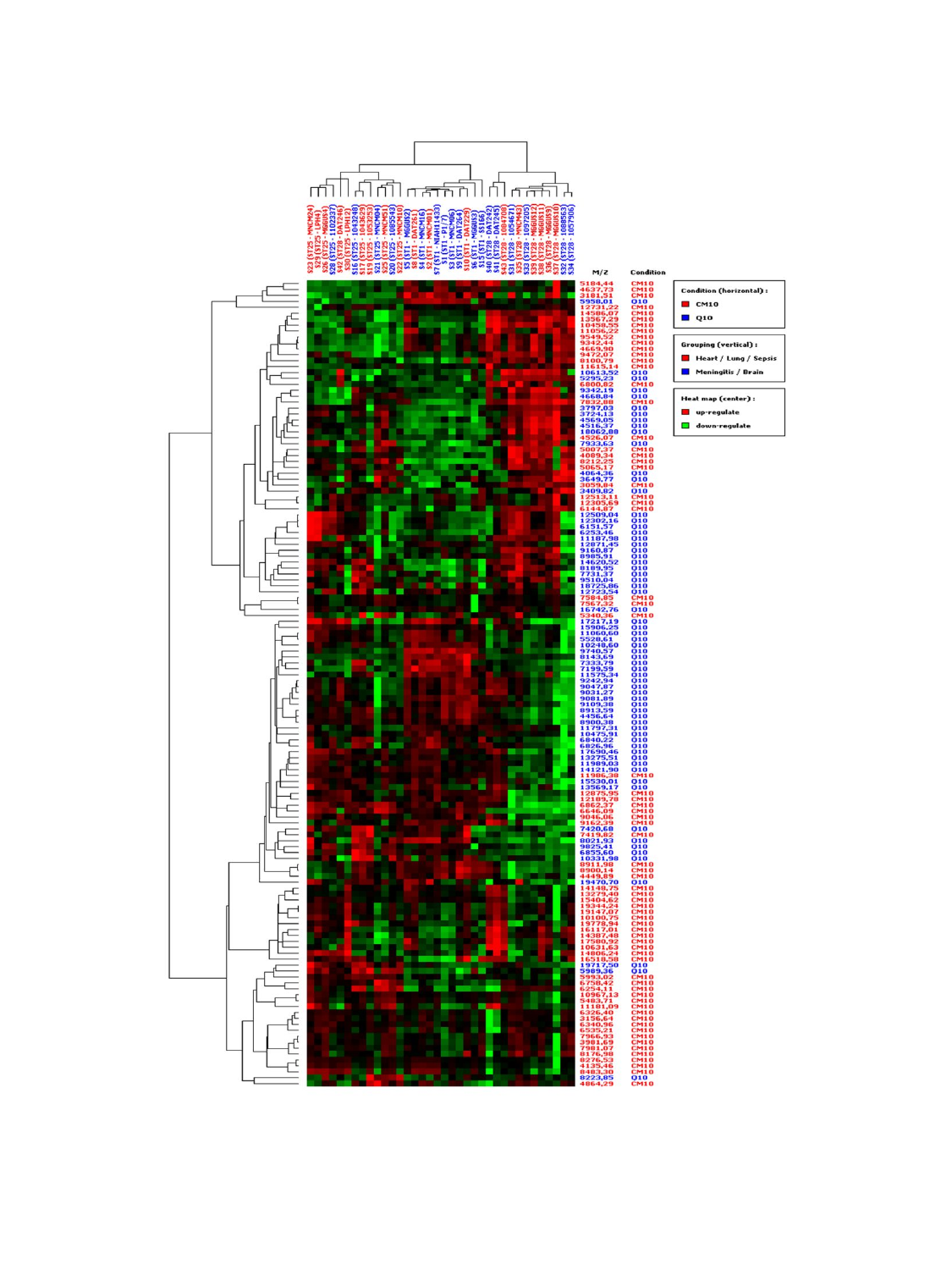

Supplement: Additional file 3: Figure S3. — Protein profiles of S. suis strains divided by type of infection/pathological lesion: group A (endocarditis/pneumonia/septicemia) versus group B (meningitis). [file 12866_2015_401_MOESM3_ESM.pptx]
